# Intrahepatic Tissue Implantation Represents a Favorable Approach for Establishing Orthotopic Transplantation Hepatocellular Carcinoma Mouse Models

**DOI:** 10.1371/journal.pone.0148263

**Published:** 2016-01-29

**Authors:** Quan Rao, Abin You, Zhenglong Guo, Bingfeng Zuo, Xianjun Gao, Ti Zhang, Zhi Du, Chenxuan Wu, HaiFang Yin

**Affiliations:** 1 Department of Cell Biology and Research Centre of Basic Medical Science, Tianjin Medical University, Qixiangtai Road, Heping District, Tianjin, 300070, China; 2 Third Central Clinical College, Tianjin Medical University, Jintang Road, Hedong District, Tianjin, 300170, China; 3 Tianjin Cancer Hospital, Tianjin Medical University, Huanhu West Road, Hexi District, Tianjin, 300000, China; 4 Department of Hepatobiliary Surgery, Key Laboratory of Artificial Cell, Institute for Hepatobiliary Diseases, Third Central Hospital, Tianjin Medical University, Jintang Road, Hedong District, Tianjin 300170, China; University of Navarra School of Medicine and Center for Applied Medical Research (CIMA), SPAIN

## Abstract

Mouse models are commonly used for studying hepatocellular carcinoma (HCC) biology and exploring new therapeutic interventions. Currently three main modalities of HCC mouse models have been extensively employed in pre-clinical studies including chemically induced, transgenic and transplantation models. Among them, transplantation models are preferred for evaluating *in vivo* drug efficacy in pre-clinical settings given the short latency, uniformity in size and close resemblance to tumors in patients. However methods used for establishing orthotopic HCC transplantation mouse models are diverse and fragmentized without a comprehensive comparison. Here, we systemically evaluate four different approaches commonly used to establish HCC mice in preclinical studies, including intravenous, intrasplenic, intrahepatic inoculation of tumor cells and intrahepatic tissue implantation. Four parameters—the latency period, take rates, pathological features and metastatic rates—were evaluated side-by-side. 100% take rates were achieved in liver with intrahepatic, intrasplenic inoculation of tumor cells and intrahepatic tissue implantation. In contrast, no tumor in liver was observed with intravenous injection of tumor cells. Intrahepatic tissue implantation resulted in the shortest latency with 0.5cm (longitudinal diameter) tumors found in liver two weeks after implantation, compared to 0.1cm for intrahepatic inoculation of tumor cells. Approximately 0.1cm tumors were only visible at 4 weeks after intrasplenic inoculation. Uniform, focal and solitary tumors were formed with intrahepatic tissue implantation whereas multinodular, dispersed and non-uniform tumors produced with intrahepatic and intrasplenic inoculation of tumor cells. Notably, metastasis became visible in liver, peritoneum and mesenterium at 3 weeks post-implantation, and lung metastasis was visible after 7 weeks. T cell infiltration was evident in tumors, resembling the situation in HCC patients. Our study demonstrated that orthotopic HCC mouse models established via intrahepatic tissue implantation authentically reflect clinical manifestations in HCC patients pathologically and immunologically, suggesting intrahepatic tissue implantation is a preferable approach for establishing orthotopic HCC mouse models.

## Introduction

Hepatocellular carcinoma (HCC) represents a global challenge due to its high morbidity and mortality rate [1]. Although intensive research has been rigorously undertaken for therapeutic interventions, a complete cure remains elusive [2]. Animal models play an indispensable role in unveiling cancer biology and contributing to the development of novel therapies. In the past few decades, a number of different HCC animal models have been established including spontaneous or drug-induced beagle dogs, cynomolgus macaques, marmosets [3] and rodents [4,5]. Among them, murine HCC models have been widely employed in HCC pre-clinical studies given the high breeding capacity, low maintenance cost, ease of handling and genetically malleability.

Currently, a variety of experimental HCC mouse models are in use for pre-clinical studies including chemically induced, transgenic and transplantation HCC mouse models [4–6]. Each model bears unique features and drawbacks, but the murine transplantation model has gradually become a mainstream for drug evaluation due to its rapid tumor occurrence. More importantly, the availability of immune-compromised nude and SCID mice allows experimentation of human cells or tissues in mice, which further favors the use of murine transplantation models [7–9].

Ectopic and orthotopic transplantation mouse models have both been extensively used in pre-clinical studies. Orthotopic mouse models are better at replicating the complex tumor-host interaction and pathological features in human, thus have been widely used to explore new antitumor therapies [10]. However, a number of different approaches are currently employed and a comprehensive comparison of these approaches will help researchers select the best approach for their experiments. Thus, we systemically compared four different approaches commonly used and characterized each model by assessing the tumor latency period, take rates, pathological features and metastatic capacity. Intravenous, intrahepatic and intrasplenic inoculation of HCC cells and intrahepatic tissue implantation were investigated side-by-side in a syngeneic setting [10–15]. The results showed that intrahepatic tissue implantation yielded more uniform and measurable solitary tumor nodules with 100% transplantability and pathological manifestations similar to HCC patients. In clear contrast, no sign of tumors was found in liver with intravenous injection of HCC cells. Although intrahepatic and intrasplenic inoculation of HCC cells resulted in tumor formation in liver, tumors were dispersed throughout with multiple nodules and lacked a well-demarcated border. Our results indicated that intrahepatic tissue implantation represents a more favorable approach for establishing orthotopic transplantation HCC mouse models.

## Materials and Methods

### Mice

6–8 weeks-old *C57BL/6* wild-type and *BALB/C* nude mice were used in all experiments (number of animals used is specified in Table 1). All the animal experiments were carried out in strict accordance with the recommendations in the Guide for the Care and Use of Laboratory Animals of Tianjin Medical University. All the procedures were authorized and approved by the committee on the Ethics of Animal Experiments of Tianjin Medical University (Permit Number: SYXK 2009–0001). All efforts were made to minimize suffering. Mice were killed by cervical dislocation at desired time-points.

**Table 1:** Tumor take rates in different models.

different approaches	Tumor take rates in different organs			
	liver	lung	spleen	heart
Intravenous injection	0/6	6/6	0/6	5/6
Intrahepatic inoculation	10/10	1/10	0/10	0/10
Intrasplenic inoculation	6/6	1/6	6/6	0/6
Tissue implantation (*C57BL6*)	49/49	2/8	0/49	0/49
Tissue implantation (Nude mice)	22/25	0/25	0/25	0/25

### Cell lines

Mouse HCC cell line Hepa1-6 (H-2^b^) was purchased from Boster Biological Technology, Ltd (Wuhan, China) and cultured in DMEM medium with 2 mM Glutamine and 10% FBS as per manufacture’s instructions. Human HCC cell line HepG2 was purchased from ATCC biobank and cultured as per manufacture’s instructions.

### Hematoxylin and eosin (H&E) staining

Routine H&E staining was used to examine the morphology and pathology of liver, spleen, lung and other tissues from HCC mouse models. Briefly, tissues were fixed in Bouin’s solution (Sigma, US) and embedded with paraffin followed by staining with hematoxylin and eosin.

### Establishment of subcutaneous HCC mouse models

Hepa1-6 or HepG2 cells were digested with 0.25% trypsin in the logarithmic phase. 3x10^6^ Hepa1-6 or HepG2 cells were suspended in 50 μl PBS and injected into left axilla of *C57BL6* or *BALB/C* nude mice subcutaneously with 1 ml syringe. Tumor was measured for longitudinal (a) and lateral (b) diameters twice a week with a vernier caliper. Tumor volume (cm^3^) was calculated with the equation of tumor volume = 1/2ab^2^.

### Establishment of orthotopic HCC mouse models by intrahepatic or intrasplenic inoculation of HCC cells

Mice were anesthetized with isoflurane and skin was sterilized with iodophor three times before surgery. For the intrahepatic inoculation of HCC cells, the surgery was conducted on left lobes along the left rib edge. 2x10^6^ Hepa1-6 cells were suspended in 50 μl PBS and injected into left lobes of the liver with a syringe at 30° angle. The injection site was gently pressed with cotton balls to reduce bleeding and leakage of cell suspensions afterwards. Then, the peritoneum and skin were closed with 4–0 sutures. For intrasplenic inoculation of HCC cells, the spleen was exposed and the same number of Hepa1-6 cells prepared as above was injected into spleen parenchyma in parallel to the long axis of spleen of the recipient mice. For intravenous injection, 2x10^6^ cell suspensions were prepared as described above and injected from tail vein.

### Establishment of orthotopic HCC mouse models by tissue implantation

Subcutaneous tumors with a longitudinal diameter of 1 cm were peeled from subcutaneous mouse models after schedule 1 killing. Tumor tissues were washed in D-hanks buffer. Necrotic tissues were removed from tumors and tumor tissues were cut into about 1 mm^3^ pieces. 2–3 tumor pieces were implanted in the left lobe of liver in the recipient mice under anesthesia.

### Measurement of CD4+/CD8+ T lymphocytes in mouse serum

Blood from orthotopic HCC mice was collected with 1% heparin, followed by lysis with the ammonium chloride-potassium (ACK) lysis buffer for 5 min in room temperature to generate lymphocyte suspensions. Mixture of lymphocytes was stained with rat anti-mouse monoclonal antibodies including PE-CD3+, percp-cy5.5 CD4+ and PE-cy7 CD8+ (ebioscience, US) at 4°C for 45 min, followed by flow cytometry analysis.

### Immunohistochemistry

To examine the presence of T lymphocytes and regulatory T cells in tumors, mouse tumors and human HCC tissues (kindly provided by biobank from Cancer Institute and Hospital of Tianjin Medical University) were fixed with Bouin’s solution (Sigma, US) and embedded with paraffin followed by staining with CD3 rabbit polyclonal Ab (Novas, US) and Foxp3+ rabbit polyclonal Ab (abcam, UK) with a dilution of 1 in 250, detected with goat-anti-rabbit secondary Ab.

### Magnetic resonance imaging (MRI)

The magnetic resonance images of orthotopic HCC mice were acquired using a 3.0 Tesia MR scanner (Signa Excite HDx; GE healthcare, Milwaukee, WI, USA) with a small animal coil in Tianjin Medical University General Hospital. The examination time-points were from 2 to 4 weeks after tissue implantation or intrahepatic inoculation of Hepa1-6 cells. During the examination, mice were anesthetized with pentobarbital sodium and fixed to minimize body motion. Coronal T2-weighted images were acquired with the following parameters: T2 propeller sequence, slice thickness of 1.0mm, slice spacing of 0.5mm, TR/TE of 3494/70.7ms, matrix of 256x160 and FOV of 8x8 cm.

### ELISA

For the cytokine assay, mouse serum was harvested from orthotopic tumor-bearing mice and centrifuged at 3000 g for 30 min at room temperature, followed by measurement of IFN-γ (R&D systems, US) and IL-10 (MultiSciences Biotech Co., Ltd., China), respectively.

### Statistical analysis

All data are reported as mean values ± SEM. Statistical differences between treatment and control groups were evaluated by SigmaStat (Systat Software, London, UK) and Kruskal-Wallis One Way Analysis of Variance on Ranks.

## Results

### Intravenous injection fails to induce tumor formation in liver

**Fig 1:**
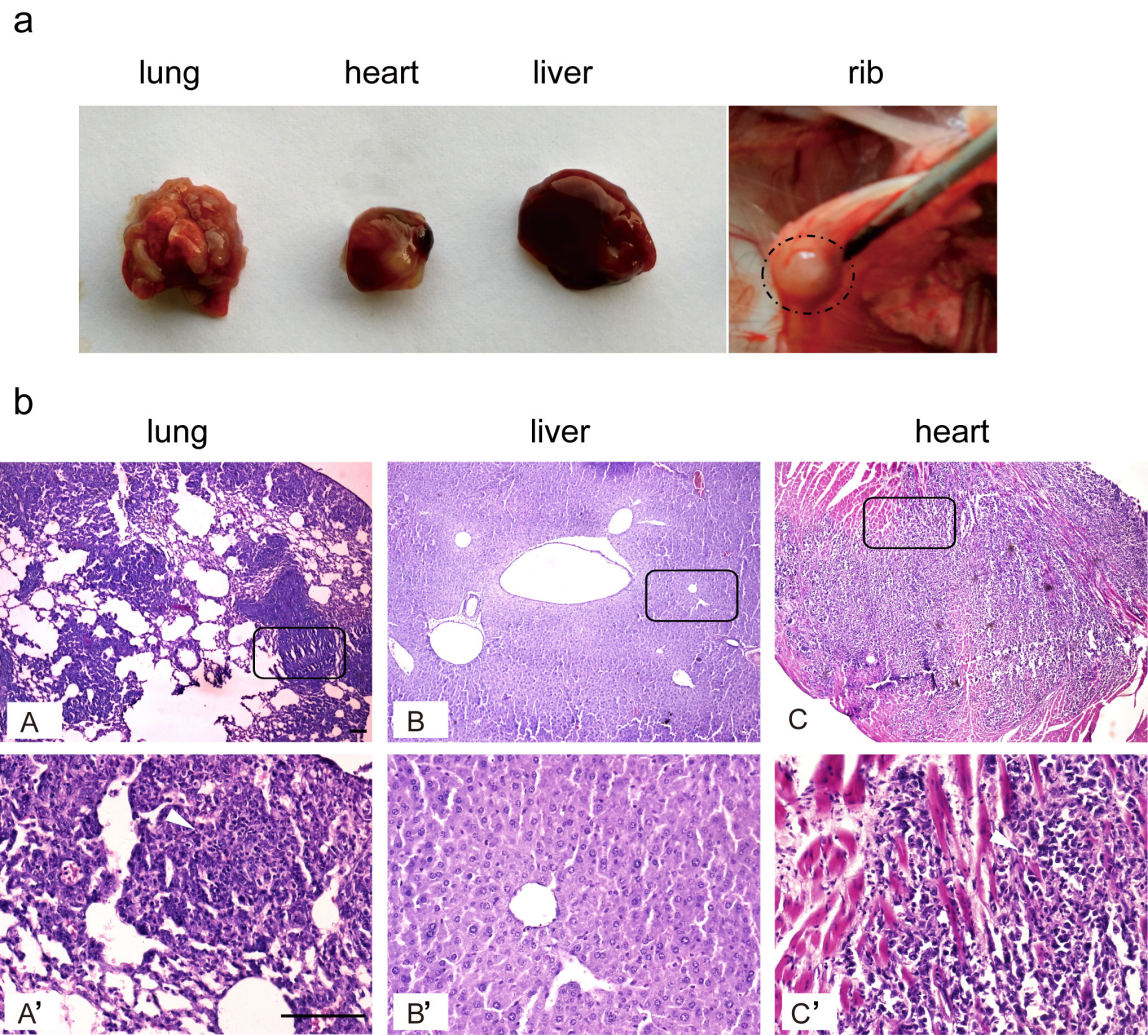
Systemic injection of Hepa1-6 cells in syngeneic *C57BL6* mice. Hepa1-6 cells (2x10^6^) suspended in PBS were injected into *C57BL6* mice intravenously. (a) Morphological examination of tumor nodules in different tissues. The results showed that tumor formation in lung, heart and ribs. (b) Histological assessment of liver tumor nodules in lung, liver and heart (scale bar = 50 μm). A’, B’ or C’ represents the corresponding magnified boxed area from A, B or C.

Intravenous injection of tumor cells is the most common non-surgical approach to establish orthotopic tumors as it is the least invasive method [11,16]. Therefore we adopted this approach for syngeneic *C57BL6* mice with Hepa1-6 cells based on previous studies and injected with a single dose of 2x10^6^ Hepa1-6 cell suspensions intravenously. Four weeks later, the animals were sacrificed and body-wide tissues were examined. Surprisingly, no tumor was found in liver, although tumors were formed in the lung and heart with 100% (6/6) and 83.3% (5/6) tumor take rates, respectively (Fig 1A, Table 1), which is consistent with previous reports [16]. Tumor nodules were also detected in rib muscles with a high occurrence rate (4/6) but not in other tissues (Fig 1A). Histological assessment demonstrated that multiple nodules appeared in the lung and heart bear typical pathological features of HCC (Fig 1B). This finding suggests that intravenous injection of tumor cells does not produce orthotopic HCC, although this approach may be useful in studying metastatic HCC.

### Intrasplenic and intrahepatic inoculations contribute to dispersed multinodular tumors in liver

**Fig 2:**
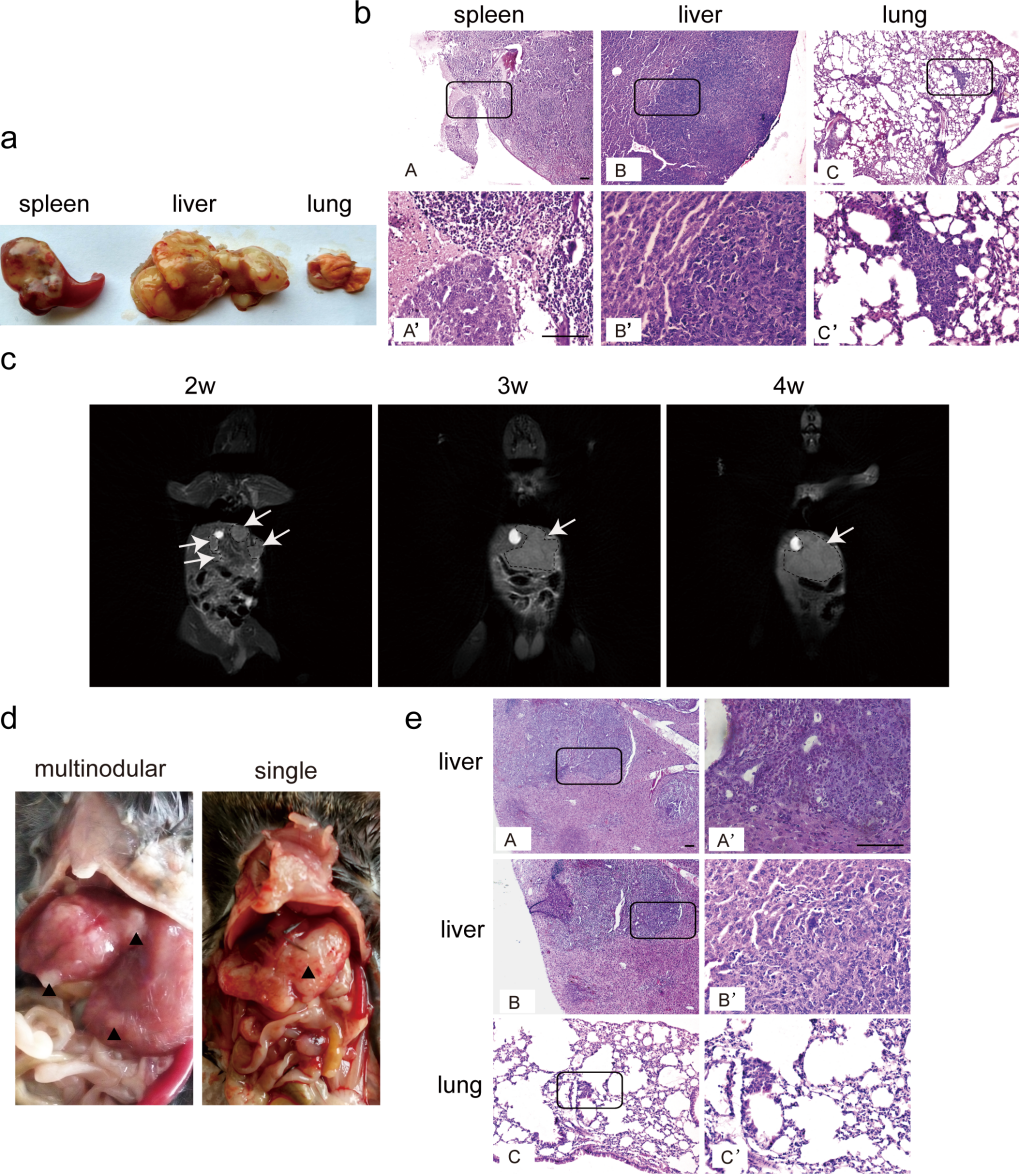
Intrasplenic and intrahepatic inoculation of Hepa1-6 cells in *C57BL6* mice. Hepa1-6 cells (2x10^6^) suspended in PBS were injected into *C57BL6* mice intrasplenicly or intrahepatically as described in Materials and Methods. (a) Morphological examination of tumor nodules in different tissues from orthotopic HCC mice generated by intrasplenic inoculation of Hepa1-6 cells. The results showed the tumor formation in liver and spleen. (b) Histological assessment of liver tumor nodules in spleen, liver and lung (scale bar = 100 μm). A’, B’ or C’ represents the corresponding magnified boxed area from A, B or C. (c) MRI analysis of the progression of liver tumors after intrahepatic inoculation of Hepa1-6 cells at different time-points. Arrows point to the tumor nodules. (d) Morphological examination of tumor nodules in liver from orthotopic HCC mice via intrahepatic injection of Hepa1-6 cells. The results showed both solitary and multinodular tumors formed in liver. (e) Histological assessment of liver tumor nodules in liver and lung (scale bar = 100 μm). A’, B’ or C’ represents the corresponding magnified boxed area from A, B or C.

We next evaluated intrasplenic and intrahepatic inoculation of tumor cells in *C57BL6* mice [17]. Some groups attempted to remove the spleen before intrasplenic inoculation [13], however as the removal of a vital immune organ can significantly impact the clinical relevance of the orthotopic model, we chose to keep the spleen intact. The same amount of Hepa1-6 cells as that used for intravenous injection was injected into the spleen parenchyma of *C57BL6* mice. Strikingly, 100% take rate was obtained in liver, though the same rate was also found in spleen 4 weeks after inoculation (Fig 2A, Table 1). However, tumors were multinodular, non-uniform and dispersed throughout liver lobes and spleen with variable sizes ranging from 0.5 to 1cm (longitudinal diameter) and were difficult to measure (data not shown). Ascites were present in some mice at 4 weeks after intrasplenic inoculation, suggesting the dispersion of tumor cells in the cavity accelerated the occurrence of ascites. Notably, tumors were also found in kidney in 1/6 mice (data not shown). Histological assessment revealed the presence of liver tumors in liver, spleen and lung, though the tumor was invisible in the lung with a low occurrence rate (1/6) (Fig 2B).

Intrahepatic injection of Hepa1-6 cells under the same dosing regimen led to 100% tumor formation in liver with 1/10 mice showing lung metastasis (Table 1). Multiple diffusive tumors became visible with variable sizes in abdominal cavity 2 weeks after injection as revealed by Magnetic Resonance Imaging (MRI) (Fig 2C). The real-time imaging demonstrated that tumors progressed rapidly from 2 to 4 weeks as shown by MRI (Fig 2C). At the 4 week time-point, multiple small tumor nodules fused and formed up to 1cm (longitudinal diameter) tumors in liver, suggesting rapid proliferation of tumor cells (Fig 2C and 2D). However, consistent with previous reports [12], dispersed, multinodular tumors of variable sizes spread throughout liver lobes, which is likely due to damage caused by the needle track (Fig 2C and 2D). 50% take rate (5/10) occurred in abdominal and pelvic cavities including peritoneum and mesenterium (data not shown), suggesting leakage contributes to the extensive tumor formation in neighboring organs. Strikingly, 2/10 mice showed solitary tumor nodule formation in liver 4 weeks after inoculation (Fig 2D). Histological examination confirmed the presence of tumors in liver and lung (Fig 2E), indicating lung metastasis occurred at 4 week time-point though with a low occurrence rate (Table 1). After 6 weeks, the majority of mice reached humane endpoints and subsequent autopsies revealed large tumors in liver and appearance of ascites, though lung metastasis was still invisible (data not shown), indicating intrahepatic inoculation of tumor cells contributes to rapid tumor progression.

### Intrahepatic tissue implantation results in uniform and measurable solitary tumor nodules in liver

**Fig 3:**
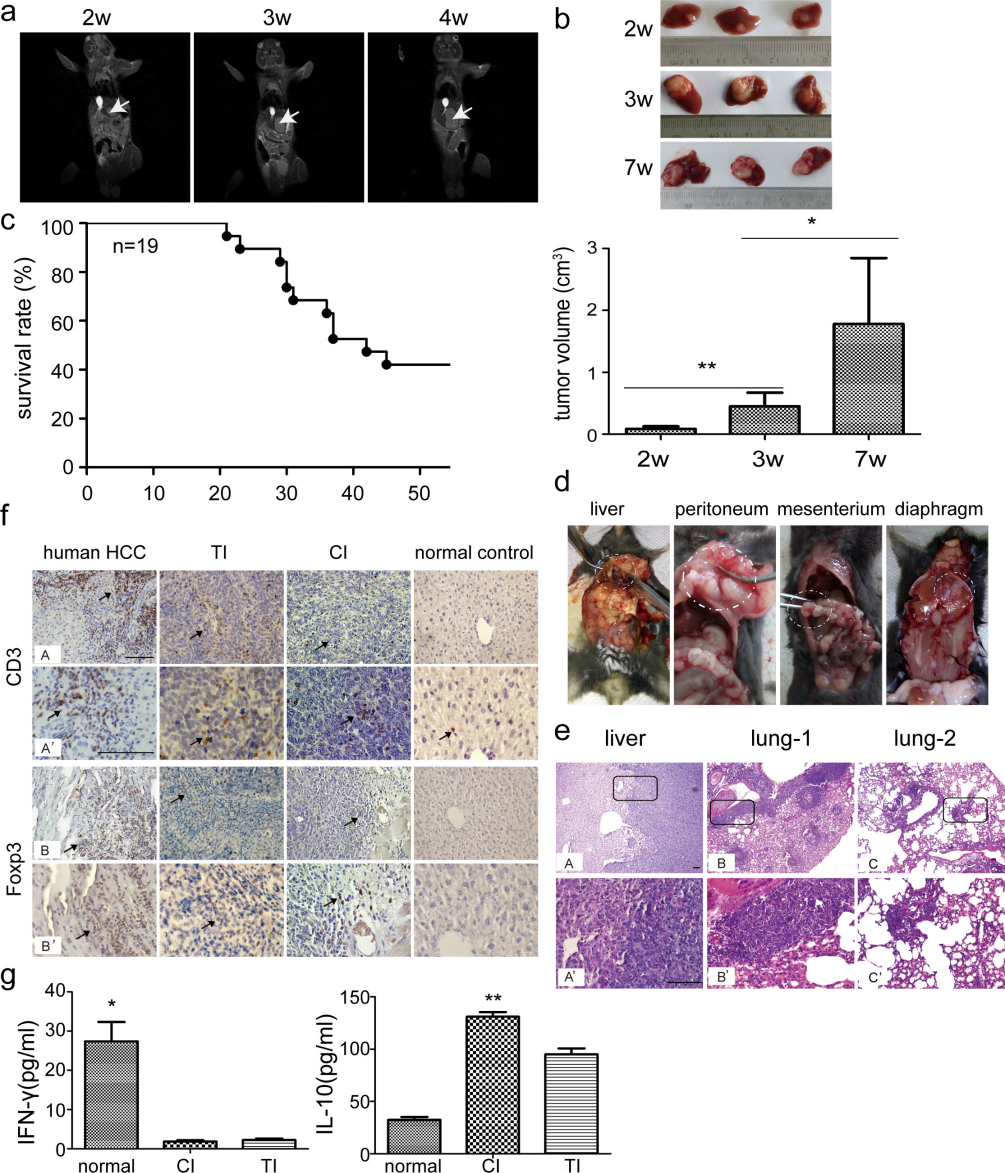
Intrahepatic tissue implantation in *C57BL6* mice. Hepa1-6 cells (3x10^6^) were suspended in 50μl PBS and injected into the left axilla of *C57BL6* mice subcutaneously with 1 ml syringe. Subsequently, tumor tissues were cut into about 1mm^3^ pieces. Tumor pieces with a size about 1mm^3^ were implanted in the left liver lobe of *C57BL6* mice. (a) MRI analysis of the progression of liver tumors after tissue implantation at different time-points. Arrows point to the tumor nodules. (b) Measurement of liver tumor sizes at different time-points after implantation. The data represents as mean±sem and significant difference was detected between different time-points (*p<0.05; **p<0.01, n = 10 for 2 week time points; n = 20 for 3 week time point; n = 19 for 7 week time point after implantation). (c) Assessment of survival rates of orthotopic HCC mice generated by intrahepatic tissue implantation (n = 19). (d) Morphological examination of tumor nodules in different tissues. The results showed the tumor formation in liver, peritoneum, mesenterium and diaphragm. (e) Histological assessment of liver tumor nodules in lung and liver (scale bar = 100 μm). A’, B’ or C’ represents the corresponding magnified boxed area from A, B or C. (f) Immunohistochemistry of CD3+ and Foxp3+ regulatory T cells in liver sections from orthotopic HCC mice and HCC patients to determine the extent of T cell infiltration (scale bar = 100 μm). Arrowhead points to CD3+ or Foxp3+ T cells. TI represents Tissue Implantation; CI denotes as Cell Inoculation. (g) Measurement of immune cytokines including IFN-γ and IL-10 in serum from orthotopic HCC mice (n = 5, *P<0.05; **P<0.01). The comparison was conducted between CI, TI and normal controls. TI represents Tissue Implantation; CI means Cell Inoculation.

Finally we investigated the tissue implantation approach in *C57BL6* mice as it is the most extensively used method for orthotopic transplantation models, given its unique advantage in preserving the integrity of tumor tissues and native cell-to-cell interaction, particularly for human tissues [7,8]. Intact tissues derived from syngenic subcutaneous tumors, originated from inoculation of Hepa1-6 cells, were implanted on the left lobe of liver in *C57BL6* mice and the animals were monitored closely. No mice died during the surgery and experiments. Uniform, solitary tumor nodules with average longitudinal diameter of 0.6±0.2cm were found in implantation sites with 100% transplantability (10/10) at 2 weeks after implantation (Fig 3A and 3B, Table 1). No sign of intrahepatic and abdominal cavity metastasis was found at this time-point after autopsy (data not shown). By 3 weeks after implantation, the tumor grew rapidly locally with a size of 0.89±0.12cm in longitudinal diameter (Fig 3A and 3B). At autopsy, intrahepatic micrometastasis (3/20) and abdominal metastasis (8/20) were present (data not shown). From week 3 to 7, local growth, regional invasion and spontaneous metastasis to liver became more evident and the tumor size reached 1.79±0.54cm in longitudinal diameter (Fig 3B). Despite extensive metastasis throughout liver lobes, about 40% tumor-bearing mice (8/19) remained alive by week 7 (Fig 3C). At autopsy, metastases were found in peritoneum, mesenterium (8/8) and diaphragm (1/8 mice) besides intrahepatic metastasis (Fig 3D), with 2/8 mice showing lung metastasis (Fig 3E). These phenotypes authentically resembled clinical manifestations in HCC patients [18], indicating that intrahepatic tissue implantation represents a favorable approach for establishing orthotopic HCC mouse models. Histological examination confirmed the presence of liver tumors in liver and lung (Fig 3E). Interestingly, only solitary tumors were produced in all the tested syngeneic *C57BL6* mice with intrahepatic tissue implantation, whereas multinodular tumors were found in nude mice when implanted with human HCC tissues, derived from subcutaneous tumors in nude mice inoculated with HepG2 cells (S1 Fig), suggesting that different genetic backgrounds of the recipient mice might affect the presentation of tumors formed.

Given the pathological similarity to HCC patients observed in orthotopic HCC mouse models, we further examined whether the orthotopic transplantation mouse model can reflect the immune microenvironment observed in HCC patients. Examination of CD3+ and Foxp3+ T lymphocytes in liver sections from orthotopic transplantation mice revealed more evident infiltration in liver tumors compared to normal liver, reminiscent of immune environment detected in HCC patients (Fig 3F). Strikingly, more CD3+ and Foxp3+ T lymphocytes were localized around tumor sites than in neighboring normal tissues in liver sections from HCC patients (Fig 3F). Measurement of CD4+ and CD8+ T lymphocytes in serum from orthotopic transplantation mice at 3 weeks after implantation revealed a decreased ratio of CD4+/CD8+ T lymphocytes compared to normal *C57BL6* mice (S2 Fig), an indicator for the extent of tumor progression [19]. Further examination of immune cytokines including interferon-γ (IFN-γ) and interleukin 10 (IL-10) in serum from orthotopic transplantation mice showed a decrease in the level of IFN-γ and an increase in the level of IL-10 (Fig 3G) compared to normal controls, suggesting that an immunosuppressive environment was formed after tumor challenge in orthotopic transplantation HCC mice. Notably, similar immunological responses were produced in HCC mice generated by intrahepatic inoculation of Hepa1-6 cells as indicated by the decreased ratio of CD4/CD8 (S2 Fig), level of IFN-γ and the increased level of IL-10 (Fig 3G). Collectively, our data demonstrated that intrahepatic tissue implantation indeed is a viable approach for establishing orthotopic transplantation HCC mice, reflecting clinical manifestations in HCC patients pathologically and immunologically.

## Discussion

A number of different approaches have been used for establishing orthotopic transplantation HCC mouse models, but a comprehensive comparison between different methods remains lacking. In this study, we investigated four commonly used approaches, namely intravenous, intrasplenic and intrahepatic inoculation of mouse HCC cells and intrahepatic tissue implantation in *C57BL6* mice [10–15]. Based on four important criteria i.e. the latency period, take rates, pathological features and metastatic rates, we systemically evaluated each approach with HCC tumor cells in a syngenic setting for the first time. Consistent with previous studies [7,20], intrahepatic tissue implantation showed superiority to other three approaches, which shows high take rate, short latency period and typical pathological features and similar metastasis pattern as manifested in HCC patients clinically. Our studies demonstrate that intrahepatic tissue implantation is the most clinically relevant approach for establishing orthotopic transplantation HCC mouse models.

Corroborating with previous reports [16], 100% transplantability was detected in lung with intravenous injection of Hepa1-6 cells in our study, though there is scarce report on the use of Hepa1-6 cells. Surprisingly, 83.3% take rate was established in heart and a lower occurrence rate in ribs with intravenous injection of Hepa1-6 cells. We cannot exclude the possibility of the unique property of HCC cells used in our study or a common phenomenon for intravenous injection of all tumor cells as intravenous injection of tumor cells is less well-documented. Nevertheless, our study confirms that intravenous injection of tumor cells is not a favorable route for establishing orthotopic HCC mouse models. Although hydrodynamic intravenous injection of tumor cells was shown to induce tumor formation in liver, lung and kidney, the technique is highly demanding [16]. More importantly, dispersed and multinodular tumors were spread throughout liver lobes, lung and kidney, which is difficult for preclinical measurement and evaluation. The same issue arose from intrasplenic inoculation of tumor cells, though 100% take rate was achieved in liver [14]. In contrast, intrahepatic inoculation of tumor cells is better than the two approaches mentioned above, though the high rate of artificial tumor dissemination contributing to the formation of diffuse and multinodular tumors and rapid progression of tumor, which shorten the lifespan of orthotopic transplantion mice.

Despite the procedures used for intrahepatic tissue implantation are slightly more complex and time-consuming than intravenous injection of tumor cells, a reproducible and stable growth of single nodule tumor makes the pre-clinical assessment possible. Importantly, orthotopic transplantation HCC mice generated by intrahepatic tissue implantation resemble clinical manifestations observed in HCC patients including pathological features, metastatic manner and immune microenvironment [7,21]. Strikingly, much shorter latency is required for intraheaptic tissue implantation compared to other three approaches. Also the lifespan of orthotopic HCC mice established via tissue implantation is much longer than counterparts generated with intrahepatic or intrasplenic inoculation of tumor cells based on our observation, which is likely attributed to the single tumor nodule formed with intrahepatic tissue implantation, rather than dispersed, multinodular tumors throughout the liver lobes produced by intrahepatic or intrasplenic inoculation, and the latter can accelerate the deterioration of liver condition and result in ascites.

In summary, we systemically evaluated four approaches commonly used in establishing orthotopic transplantation HCC mouse models and identified that intrahepatic tissue implantation is the best approach to produce clinically relevant orthotopic HCC models.

## Supporting Information

S1 Fig Intrahepatic tissue implantation of human HCC tissues in immune-compromised nude mice (*BALB/C*). (PDF)

S2 Fig Measurment of CD4+ and CD8+ T lymphocytes in serum from orthotopic mice via intrahepatic tissue implantation and intrahepatic inoculation of Hepa1-6 cells. (PDF)
