# Correction: Intrahepatic Tissue Implantation Represents a Favorable Approach for Establishing Orthotopic Transplantation Hepatocellular Carcinoma Mouse Models

**DOI:** 10.1371/journal.pone.0206322

**Published:** 2018-10-18

**Authors:** Quan Rao, Abin You, Zhenglong Guo, Bingfeng Zuo, Xianjun Gao, Ti Zhang, Zhi Du, Chenxuan Wu, HaiFang Yin

Fig 2 is incorrect. Please view the correct Fig 2 here.

**Fig 2:**
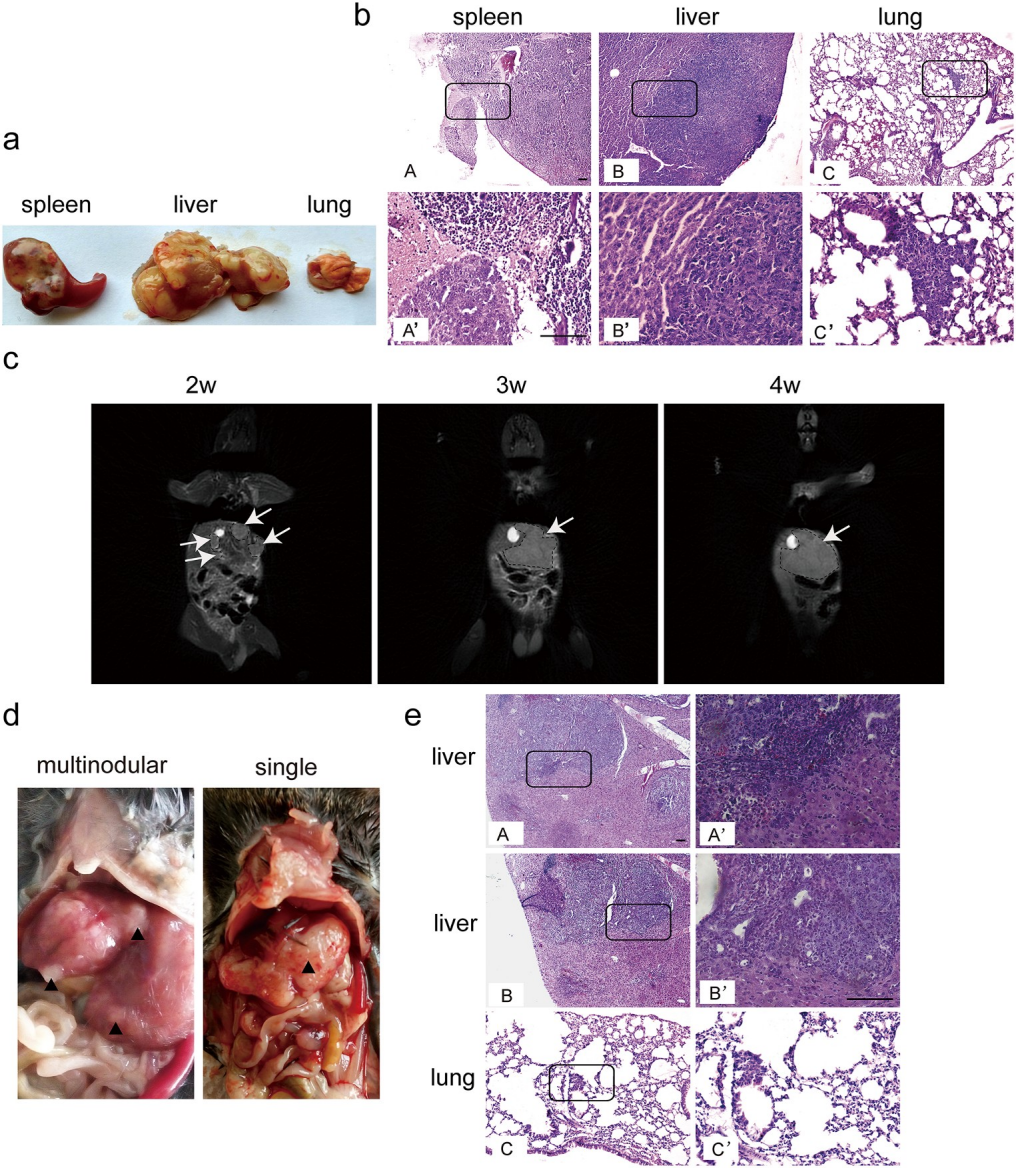
Intrasplenic and intrahepatic inoculation of Hepa1-6 cells in *C57BL6* mice. Hepa1-6 cells (2x10^6^) suspended in PBS were injected into *C57BL6* mice intrasplenicly or intrahepatically as described in Materials and Methods. (a) Morphological examination of tumor nodules in different tissues from orthotopic HCC mice generated by intrasplenic inoculation of Hepa1-6 cells. The results showed the tumor formation in liver and spleen. (b) Histological assessment of liver tumor nodules in spleen, liver and lung (scale bar = 100 μm). A’, B’ or C’ represents the corresponding magnified boxed area from A, B or C. (c) MRI analysis of the progression of liver tumors after intrahepatic inoculation of Hepa1-6 cells at different time-points. Arrows point to the tumor nodules. (d) Morphological examination of tumor nodules in liver from orthotopic HCC mice via intrahepatic injection of Hepa1-6 cells. The results showed both solitary and multinodular tumors formed in liver. (e) Histological assessment of liver tumor nodules in liver and lung (scale bar = 100 μm). A’, B’ or C’ represents the corresponding magnified boxed area from A, B or C.

## References

[1] Rao Q, You A, Guo Z, Zuo B, Gao X, Zhang T, et al (2016) Intrahepatic Tissue Implantation Represents a Favorable Approach for Establishing Orthotopic Transplantation Hepatocellular Carcinoma Mouse Models. PLoS ONE 11(1): e0148263 10.1371/journal.pone.0148263 26824903 PMC4732811

