# Supplementary material for: Intrahepatic Tissue Implantation Represents a Favorable Approach for Establishing Orthotopic Transplantation Hepatocellular Carcinoma Mouse Models
Source: PLoS One. 2016 Jan 29;11(1):e0148263. doi: 10.1371/journal.pone.0148263 (PMC4732811; doi:10.1371/journal.pone.0148263)
Supplement: S1 Fig — (PDF) [file pone.0148263.s001.pdf]

S1 Fig. Intrahepatic tissue implantation of human HCC tissues in immune-compromised nude mice (*BALB/C*).

a

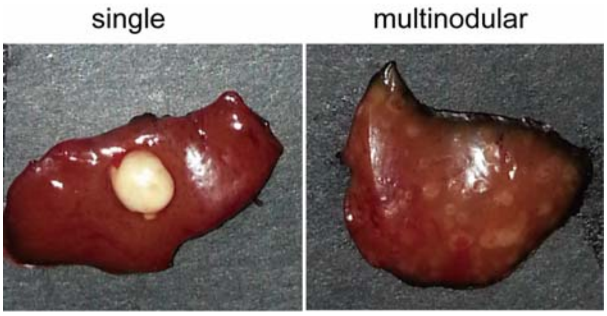

b

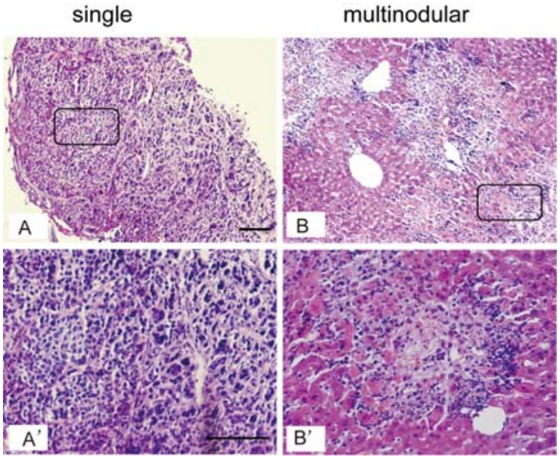

HepG2 ($3x10^6$) cells were suspended in 50 μl PBS and injected into left axilla of *BALB/C* nude mice subcutaneously with 1 ml syringe. Subsequently, tumor tissues were cut into about $1mm^3$ pieces. 2-3 tumor pieces were implanted in the left lobe of liver in the recipient mice under anesthesia. (a) Morphological examination of tumor nodules in livers. The results showed that solitary or multinodular tumors were formed in livers from *BALB/C* nude mice. (b) Histological assessment of liver tumor nodules in liver (scale bar = 100 μm). A' or B' represents the corresponding magnified boxed area from A or B.
