# Supplementary material for: Intrahepatic Tissue Implantation Represents a Favorable Approach for Establishing Orthotopic Transplantation Hepatocellular Carcinoma Mouse Models
Source: PLoS One. 2016 Jan 29;11(1):e0148263. doi: 10.1371/journal.pone.0148263 (PMC4732811; doi:10.1371/journal.pone.0148263)
Supplement: S2 Fig — (PDF) [file pone.0148263.s002.pdf]

S2 Fig. Measurment of CD4+ and CD8+ T lymphocytes in serum from orthotopic mice via intrahepatic tissue implantation and intrahepatic inoculation of Hepa1-6 cells.

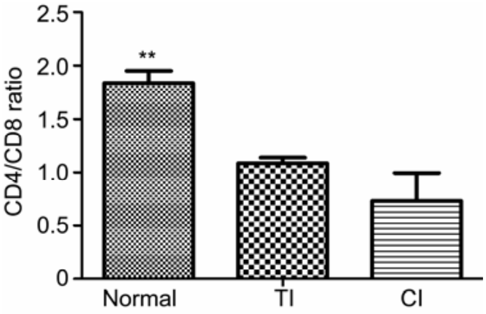

Serum was collected from orthotopic HCC mice 3 weeks after implantation or inoculation and measurement of CD4+ and CD8+ T lymphocytes revealed that the ratio of CD4+/CD8+ declined, indicating an immunosuppressive niche formed ($p<0.01$, n=5 for orthotopic HCC mice and n=3 for normal control group). TI represents Tissue Implantation; CI means Cell Inoculation.
